# Barrier mechanisms in the *Drosophila* blood-brain barrier

**DOI:** 10.3389/fnins.2014.00414

**Published:** 2014-12-16

**Authors:** Samantha J. Hindle, Roland J. Bainton

**Affiliations:** Department of Anesthesia and Perioperative Care, University of California, San FranciscoSan Francisco, CA, USA

**Keywords:** invertebrates, blood-brain barrier, chemoprotection, drug delivery, conserved physiology

## Abstract

The invertebrate blood-brain barrier (BBB) field is growing at a rapid pace and, in recent years, studies have shown a physiologic and molecular complexity that has begun to rival its vertebrate counterpart. Novel mechanisms of paracellular barrier maintenance through G-protein coupled receptor signaling were the first demonstrations of the complex adaptive mechanisms of barrier physiology. Building upon this work, the integrity of the invertebrate BBB has recently been shown to require coordinated function of all layers of the compound barrier structure, analogous to signaling between the layers of the vertebrate neurovascular unit. These findings strengthen the notion that many BBB mechanisms are conserved between vertebrates and invertebrates, and suggest that novel findings in invertebrate model organisms will have a significant impact on the understanding of vertebrate BBB functions. In this vein, important roles in coordinating localized and systemic signaling to dictate organism development and growth are beginning to show how the BBB can govern whole animal physiologies. This includes novel functions of BBB gap junctions in orchestrating synchronized neuroblast proliferation, and of BBB secreted antagonists of insulin receptor signaling. These advancements and others are pushing the field forward in exciting new directions. In this review, we provide a synopsis of invertebrate BBB anatomy and physiology, with a focus on insights from the past 5 years, and highlight important areas for future study.

## Introduction

In order for the nervous system to perform efficiently in coordinating sophisticated movements, behaviors and cognitive functions, it requires chemical isolation from the fluctuating, and potentially damaging, components in the blood. The blood-brain barrier (BBB) has evolved to provide this role, becoming more sophisticated with increasing complexity and demand of the nervous system (Abbott, 2005). Therefore, there are alternative cellular strategies for making BBB structures. In insects, crustacea, cephalopod molluscs and cartilaginous fish the BBB is formed by glial cells, whereas in higher order vertebrates the BBB is formed primarily by the brain vascular endothelium. The formation and functions of the vertebrate BBB are covered extensively by various reviews (e.g., Abbott, 2005; Abbott et al., 2010; Obermeier et al., 2013) and within this BBB series; the aim of this review is to describe the comparative compound structure and associated physiologies of the invertebrate BBB. In recent years, the invertebrate BBB field has flourished with interesting insights into the mechanisms of BBB maintenance, as well as discovering novel metabolic and signaling roles. In addition, both established and emerging invertebrate models are proving useful as drug screening tools to advance therapies for neurodegenerative diseases. These findings, and others, are providing many exciting avenues for future BBB investigations.

## Anatomical overview of the invertebrate BBB

**Figure 1:**
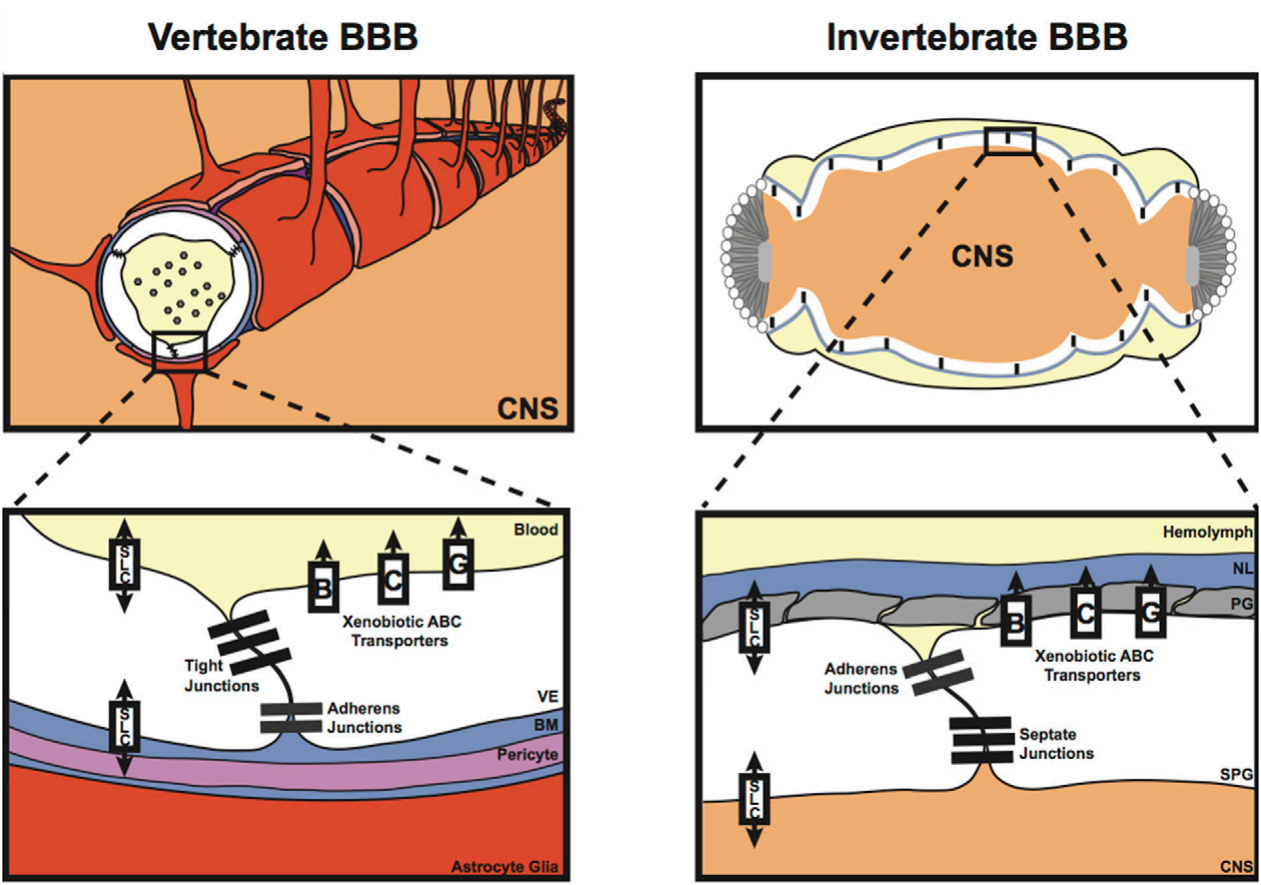
**Diagrammatical representation of the vertebrate and invertebrate blood-brain barrier (BBB)**. The vertebrate BBB is primarily formed by the vascular endothelial cells (VE) that form the capillaries in the brain; however, the surrounding pericytes within the basement membrane (BM), and the end feet of the astrocyte glia also contribute to vertebrate BBB functions. These cellular and non-cellular layers form a compound barrier structure known as the neurovascular unit (NVU). The invertebrate BBB is also a compound structure, consisting of the subperineurial glia (SPG), the perineurial glia (PG), and also a basement membrane known as the neural lamella (NL). Both the vertebrate and invertebrate barriers express tight/septate junction and adherens junction proteins, as well as various xenobiotic ATP-binding Cassette (ABC) and Solute Carrier (SLC) transporters required to maintain central nervous system (CNS) homeostasis.

**Figure 2:**
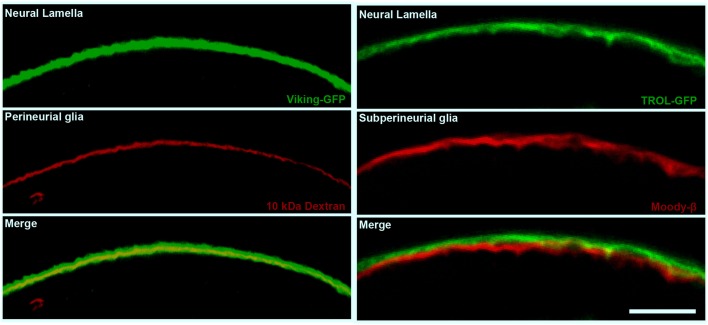
**The cellular and non-cellular layers of the *Drosophila* compound BBB**. Confocal micrographs of cross sections of the adult *Drosophila* optic lobe showing the localization of the neural lamella (green, left and right panels), the perineurial glia (PG) layer (red, left panels) and the subperineurial glia (SPG) layer (red, right panels). The left panels show the positioning of the neural lamella (identified by the Collagen IV marker Viking-GFP) with respect to the PG (marked by 10 kDa Texas Red Dextran staining). The right panels show the closely apposed neural lamella (marked by the extracellular matrix proteoglycan TROL-GFP) and SPG layer (stained by Moody-β antibody). Scale bar, 20 μm.

Unlike in vertebrates, the blood (hemolymph) in insects does not circulate in a closed capillary network; instead, the hemolymph is freely flowing in the body cavity. As a result, protection of the insect nervous system from the high and often fluctuating concentrations of hemolymph potassium requires that the BBB entirely surrounds the nervous system. Therefore, at the gross anatomical level, the invertebrate BBB is quite different from that of the vascular endothelium in vertebrates. However, like its vertebrate counterpart, the invertebrate BBB is a compound structure (Figure 1), analogous to the vertebrate neurovascular unit (Stork et al., 2008; Mayer et al., 2009; DeSalvo et al., 2011, 2014). The invertebrate compound BBB is primarily composed of two surface glia cell types [the apical perineurial glia (PG) and the basal subperineurial glia (SPG)], and the exterior extracellular matrix layer known as the neural lamella (Figures 1, 2). The absence of almost all glial cells in the *Drosophila glial cells missing* (*gcm*) mutant renders the nervous system permeable to a broad range of fluorescent dextran molecules ranging from 10 to 500 kDa (Stork et al., 2008), confirming the importance of glial cells for the invertebrate BBB.

The protective functions of the invertebrate BBB are apparent from embryonic stage 17 throughout development and into the adult stage (Bainton et al., 2005; Schwabe et al., 2005; Stork et al., 2008; Mayer et al., 2009). The PG, however, are not thought to contribute toward these BBB properties early in development as PG cells do not proliferate and completely surround the CNS until late larval stages (Stork et al., 2008). The roles of the BBB during early development are therefore attributed to the SPG, which entirely encapsulate the embryonic nervous system and maintain a tight barrier throughout development and into the adult stage (Stork et al., 2008). In order to maintain this tight diffusion barrier, the SPG cells do not proliferate as the nervous system grows; instead the SPG increase in size and become polyploid (Unhavaithaya and Orr-Weaver, 2012). The polyploid nature of the SPG is essential to maintain the integrity of the septate junctions that are present between the SPG cells, as the nervous system grows. Inhibition of polyploidy in the SPG causes the septate junctions to rupture and diffusion barrier integrity to be lost (Unhavaithaya and Orr-Weaver, 2012). The formation of the invertebrate BBB has been covered extensively elsewhere (Stork et al., 2008; Edwards and Meinertzhagen, 2010; DeSalvo et al., 2011).

The majority of research on the invertebrate BBB has focused on the main BBB layer, the SPG. However, recent investigations have started to uncover additional roles for cellular and non-cellular layers external to the SPG cells. When the septate junction protein Neurexin IV was mutated in *Drosophila*, the brain penetration of 500 kDa dextran was delayed compared to in the *glial cells missing* mutant, which lacks almost all glial cells (Stork et al., 2008). This suggests that barriers other than the SPG septate junctions are present that can reduce the brain access of certain high molecular weight molecules. It is therefore possible that the PG and neural lamella layers act as non-specific, large molecular-weight filters. Moreover, DeSalvo et al. (2011, 2014) and Meyer et al. (2014) have recently suggested that the PG may have signaling and metabolic roles in BBB function, and in maintaining the neural lamella. Collagen IV, which forms a major part of the neural lamella, is secreted by hemocytes in the embryo and the fat body during post-embryonic development (Mirre et al., 1988; Pastor-Pareja and Xu, 2011); but Meyer et al. (2014) showed that, in addition to the fat body, the *Drosophila* BBB can also contribute to the integrity of the neural lamella. Mutations that affect the deposition of Collagen IV into the neural lamella, result in deformation of the central nervous system (CNS) and deficits in nervous system function (Olofsson and Page, 2005; Meyer et al., 2014). Knocking out matrix metalloproteinases specifically in the SPG or PG cells resulted in larval lethality or an extended ventral nerve cord phenotype, respectively (Meyer et al., 2014). This suggests that the BBB glia are required to maintain the structural integrity of the lamella, which is necessary for correct CNS shape. It therefore appears that the glial cells of the BBB may coordinate with the fat body to maintain and restructure the neural lamella to respond to changing developmental and possibly metabolic demands.

In addition to coordination between the BBB and fat body for maintenance of BBB functions, evidence suggests that neurons also have a role to play. Rbp9, which is homologous to the RNA-binding proteins elav (invertebrates) and hu (vertebrates), has been shown to function in maintaining BBB integrity; loss of Rbp9 function leads to down-regulation of septate junction proteins (primarily Neurexin IV and Coracle), and a resultant porous BBB (Kim et al., 2010). Interestingly, Rbp9 is expressed in neurons, but not in glia, and is therefore not expressed in the BBB itself (Kim-Ha et al., 1999). As the *Rbp9* mutants initially form an intact BBB and only show a porous BBB phenotype upon adult eclosion (Kim et al., 2010), it appears that neuronal Rbp9 plays an important role in BBB maintenance in invertebrates, analogous to the involvement of neuronal signaling in vertebrate BBB integrity (Stewart and Wiley, 1981; Stenman et al., 2008; Daneman et al., 2009). In conclusion, it appears that, like the vertebrate neurovascular unit, there is an important involvement of all layers of the invertebrate compound BBB in the formation and maintenance of the BBB.

## Molecular overview of the invertebrate BBB

As was mentioned above, the vertebrate vascular endothelium of the brain is specialized to provide an extremely tight barrier to transcellular and paracellular molecular transit. This is due to the following vascular endothelium properties: tight junction complexes between the vascular endothelium cells; enrichment of efflux ABC transporters, which are localized to the apical (luminal) face of the vascular endothelium; low rates of trancytosis due to reduced vesicular trafficking (Muir and Peters, 1962; Reese and Karnovsky, 1967; Brightman and Reese, 1969; Daneman et al., 2010; for reviews see Zlokovic, 2008; Abbott et al., 2010; Obermeier et al., 2013). Many of these molecular properties are also shared by the invertebrate BBB. As alluded to above, the SPG cells of the invertebrate BBB form a tight barrier to paracellular transit due to the presence of pleated septate junctions. These complexes are equivalent to the septate junctions that perform electrical and chemical insulation properties at the axo-glial paranodal junctions (Bhat et al., 2001; Bhat, 2003). SPG cells also express efflux ABC transporters, in particular the ABC B1 xenobiotic efflux transporter Mdr1/P-glycoprotein homolog (Mdr65) is both highly enriched and shows apical localization in the SPG cells (Mayer et al., 2009; DeSalvo et al., 2014). In addition to the chemoprotective properties, the BBB must also regulate the brain access of endogenous molecules, such as glucose and ions. For this, the BBB expresses various solute carrier proteins (SLCs). Recent evidence from *Drosophila* has also suggested interesting roles for the gap junctions that are expressed in both the vertebrate and invertebrate BBB. These findings are discussed in more detail below.

### Chemoprotective roles of the invertebrate BBB

#### Septate junction formation and maintenance

**Figure 3:**
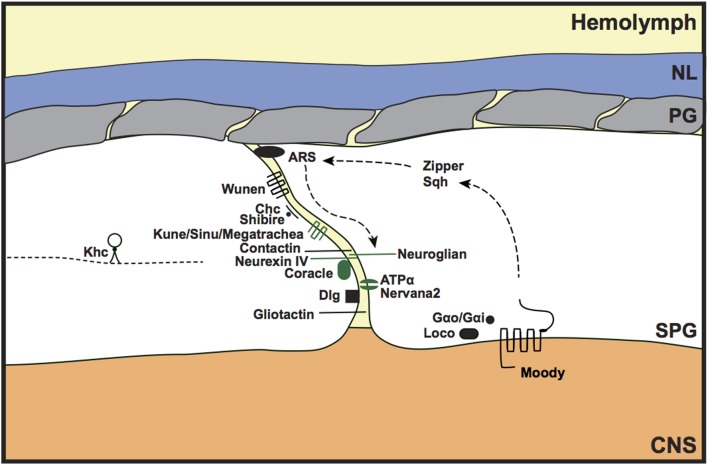
**Septate junction formation and maintenance in the invertebrate BBB**. Components of the core septate junction (green) and the associated proteins that maintain BBB integrity (black) were identified from loss of function mutants that led to a porous BBB phenotype. *Discs large (Dlg)* mutants showed delayed assembly of the core septate junction components, whereas mutants of Gliotactin, and the endocytic proteins Clathrin-heavy chain (Chc) and Shibire, showed a mislocalization of the septate junction core. The Moody GPCR signaling pathway (Moody, G_α*o*_/G_α*i*_, loco) was shown to genetically interact with actin and non-muscle myosin components [actin-rich structures (ARS), Zipper, Spaghetti squash (Sqh)] to maintain septate junction integrity. Kinesin heavy chain (Khc) may regulate septate junction integrity by transporting Neurexin IV as a cargo protein. Loss of septate junction integrity by disrupting the function of wunen, the lipid phosphate phosphatase, suggests a role for bioactive lipids in paracellular barrier function. NL, neural lamella; PG, perineurial glia; SPG, subperineurial glia; CNS, central nervous system; kune (kune-kune).

The primary barrier of the invertebrate BBB is provided by the septate junctions, the functional equivalent of the vertebrate tight junctions and paranodal septate junctions. The formation and maintenance of a tight diffusion barrier protects the invertebrate nervous system from the high concentrations of potassium in the hemolymph; without this barrier, the embryos are unable to fire action potentials and cannot coordinate movements to hatch from the embryonic cuticle. Septate junction mutations therefore cause a porous BBB and embryonic lethality (e.g., Baumgartner et al., 1996; Schwabe et al., 2005; Stork et al., 2008). The 5 main components of the septate junctions are the membrane components Neurexin IV (Caspr in vertebrates), Neuroglian (vertebrate Neurofascin 155), ATPα and Nervana2 (Na^+^/K^+^ ATPase α and β subunits), and the intracellular component Coracle (protein 4.1 in vertebrates); however additional proteins are also thought to contribute toward the integrity and maintainance of the septate junctions, including Gliotactin (vertebrate Neuroligin-like), Contactin (vertebrate Contactin), Discs large (Dlg; vertebrate PSD-95-like), Fasciclin III, Moody, Sinuous, and Megatrachea (the latter 2 are vertebrate claudin-like proteins) (Fehon et al., 1994; Auld et al., 1995; Baumgartner et al., 1996; Behr et al., 2003; Genova and Fehon, 2003; Paul et al., 2003; Schulte et al., 2003; Faivre-Sarrailh et al., 2004; Bainton et al., 2005; Schwabe et al., 2005; Stork et al., 2008; Oshima and Fehon, 2011). The septate junction proteins interact with each other to form stable septae contacts between SPG cells that surround the CNS (Figure 3). Disruption of any septate junction component leads to a porous BBB phenotype, suggesting that they function interdependently to maintain BBB function. To further address the relationship between septate junction components, Oshima and Fehon (2011) investigated the dynamics of septate junction-associated proteins. Using fluorescence recovery after photobleaching (FRAP), they elegantly showed that the 5 main septate junction components form a very stable core unit, whereas other septate junction-associated proteins (e.g., Dlg) are more mobile. In addition, they discovered that mutating different septate junction-associated proteins had different mechanisms for causing a defective BBB. Loss of some septate junction-associated proteins (e.g., Dlg) caused delayed formation of the stable septate junction core; however, the dynamics of this stable core, once formed, was not affected by *Dlg* mutation. Furthermore, the loss of Gliotactin or endocytic proteins, such as clathrin heavy chain or the *Drosophila* dynamin homolog shibire, led to a mislocalisation of the core septate junction proteins, but had no effect on septate junction core formation (Oshima and Fehon, 2011).

Interestingly, a particular splice form of Neurexin IV (containing exon 3) was shown to be the septate junction-forming version of Neurexin IV and has been shown to be preferentially expressed in glia over neurons (Rodrigues et al., 2012). The mechanism of cell-specific splicing has recently been elucidated in *Drosophila* using elegantly designed genetic reagents that revealed the transcript pattern of the Neurexin IV isoform(s) present in neurons vs. glia (Rodrigues et al., 2012). The authors found that the cell-specific splicing of Neurexin IV involves the RNA-binding protein HOW (Held out wings). HOW is not expressed in neurons, but ectopic neuronal expression of the constitutively nuclear form of HOW leads to a shift in Neurexin IV isoform expression to that of a glial pattern, leading to larval lethality. This showed that the expression and nuclear localization of HOW in glia predisposes glia to expression of the Neurexin IV (exon 3) form, promoting the formation of septate junctions. Rodrigues et al. (2012) showed the additional involvement of the kinase Cdk12 and the splicesomal component Prp40 in HOW function.

Recently, another claudin-like protein (Kune-kune) and a leukocyte antigen 6 family protein (Coiled) were identified in screens for disrupted BBB integrity (Nelson et al., 2010; Hijazi et al., 2011; Syed et al., 2011). The protein Kune-kune colocalizes with the septate junction component Coracle. Its localization is dependent on the presence of various septate junction components (e.g., Coracle, ATPα, Sinuous and Megatrachea) and, reciprocally, septate junction component localization is dependent on the presence of Kune-kune (Nelson et al., 2010). The authors discovered that Kune-kune regulates both septate junction localization and levels, whereas the other claudin-like proteins Sinuous and Megatrachea regulate septate junction component levels and localization, respectively. Analysis of septate junction levels and localization in single, double and triple mutants of these three claudin-like proteins showed that Kune-kune seems to have a more important role in coordinating septate junction components than Sinuous and Megatrachea (Nelson et al., 2010). The Ly6 family protein Coiled is required for septate junction formation in the embryo; *Coiled* mutations cause disrupted localization of septate junction proteins, including Neurexin IV, Coracle and Discs large, therefore abrogating BBB integrity (Hijazi et al., 2011; Syed et al., 2011).

The G protein-coupled receptors (GPCRs) encoded by the *moody* gene (Moody-α and Moody-β) are required to regulate BBB permeability (Bainton et al., 2005; Schwabe et al., 2005). In addition, the trimeric G proteins Gαi and Gαo, and the regulator of G protein signaling (RGS) loco are thought to signal in the same Moody pathway to regulate septate junction formation and maintenance (Schwabe et al., 2005). Mutations in any of these four components leads to a porous BBB and locomotor dysfunction; however, the *moody* null has a weaker BBB permeability to 10 kDa dextran compared to the *Gαi, Gαo*, and *loco* mutants, suggesting that these latter components of the GPCR signaling pathway may have additional upstream or downstream signaling partners involved in septate junction integrity. Interestingly, because loco is a negative regulator of GPCR signaling, this means that both loss and gain of GPCR signaling disrupts BBB integrity. This suggests that discreet, and localized control over this signaling pathway is required for correct septate junction formation and maintenance. That being said, the effect on septate junction integrity appears to be a secondary effect of disrupted GPCR signaling on the actin cytoskeleton and cell size/shape, which leads to reduced undulations of the SPG cells and a shortening of the septate junction length (Schwabe et al., 2005). As the length of the septate junction dictates the tightness of the BBB, this creates a more permeable septate junction barrier in *moody* mutants.

Additional evidence that BBB integrity requires the coordination of Moody signaling and actin dynamics was recently provided by Hatan et al. (2011). They discovered that highly dynamic actin-rich structures (ARSs) are localized in close proximity to, but not overlapping with, the septate junctions; the ARSs colocalized with the fusion proteins Neuroglian-GFP and Scribble-GFP, but not with Neurexin-IV. The regulation of ARS morphology and size was shown to require Moody signaling and also activation of non-muscle myosin components Zipper and Spaghetti squash, which are thought to be downstream of Moody signaling (Hatan et al., 2011). Disruption of ARSs by knocking down the actin nucleation components Arp2 and Arp3 led to dissociation of ARSs and Neuroglian, discontinuous Neuroglian labeling at the septate junction, and breakdown of BBB integrity. These findings suggest an interesting possibility that ARSs act as dynamic docking sites for septate junction components to allow their rapid incorporation into the septate junctions during development to maintain a tight BBB as the brain grows (Figure 3). Although the ARSs were only detectable during development, and not during adult stages (Hatan et al., 2011), it would be interesting to determine whether similar structures have a transitory role in the adult, perhaps during circumstances of BBB challenge (chemical, physical or pathological).

Another regulator of BBB integrity, the Kinesin heavy chain, was recently identified from a 5000 candidate-gene screen for locomotion defects (Schmidt et al., 2012). Kinesin heavy chain is classically known for its role in axonal transport. However, Schmidt et al. (2012) discovered an important role within SPG cells. Knocking down *Kinesin heavy chain* specifically in SPG cells led to disrupted axonal excitability resulting in spasticity in adult flies. They found that one of the cargoes of a Kinesin heavy chain-dependent Rab protein is Neurexin-IV, suggesting that Kinesin heavy chain may contribute toward the control of neuronal excitability by regulating Neurexin-IV incorporation into the septate junctions of the SPG cells. The vertebrate homologs of *Drosophila* Kinesin heavy chain are members of the KIF5 family. Both KIF5A and KIF5C are mainly expressed in neurons, but KIF5B shows some expression in glia (Kanai et al., 2000); however, no specific roles have been found for KIF5B in glia. Interestingly, the kinesin KIF13B has been shown to transport Discs large 1 (Dlg1) to sites of membrane remodeling during myelin formation in the Schwann glial cells that insulate the peripheral nervous system (Bolis et al., 2009). Therefore, homologous roles may exist for Kinesin heavy chain proteins in regulating tight junction integrity in the vertebrate BBB.

Wunen is a lipid phosphate phosphatase (LPP), an integral membrane enzyme that, in vertebrates, is required in the regulation of bioactive lipids (e.g., sphingosine-1-phosphate). In *Drosophila*, Wunen is known for its role in germ cell survival and migration (Zhang et al., 1997; Starz-Gaiano et al., 2001); however, it has recently been shown to also localize to septate junctions and to function in septate junction maintenance both in trachea and the BBB (Ile et al., 2012; Figure 3). *Wunen* mutant nerve cords show increased permeability to 10 kDa dextran, comparable to that of a Neurexin-IV mutant (Ile et al., 2012). A role for bioactive lipids in the maintenance of BBB integrity has previously been show in mammalian brain microvascular endothelial cells grown in culture (Lee et al., 2006). Treatment with sphingosine-1-phosphate caused relocalization of tight junction/cell-cell contact proteins (including ZO-1 and claudin-5) and increased the trans-epithelial electrical resistance. Also, an *in vivo* role for the mammalian LPP3 in the formation of vascular endothelial cell interactions was shown during development (Escalante-Alcalde et al., 2003). This shows that the role for bioactive lipids in BBB formation and maintenance is evolutionarily conserved, and supports the notion that the invertebrate septate junctions are functionally and mechanistically equivalent to the vertebrate tight junctions.

#### Xenobiotic efflux transporters

**Figure 4:**
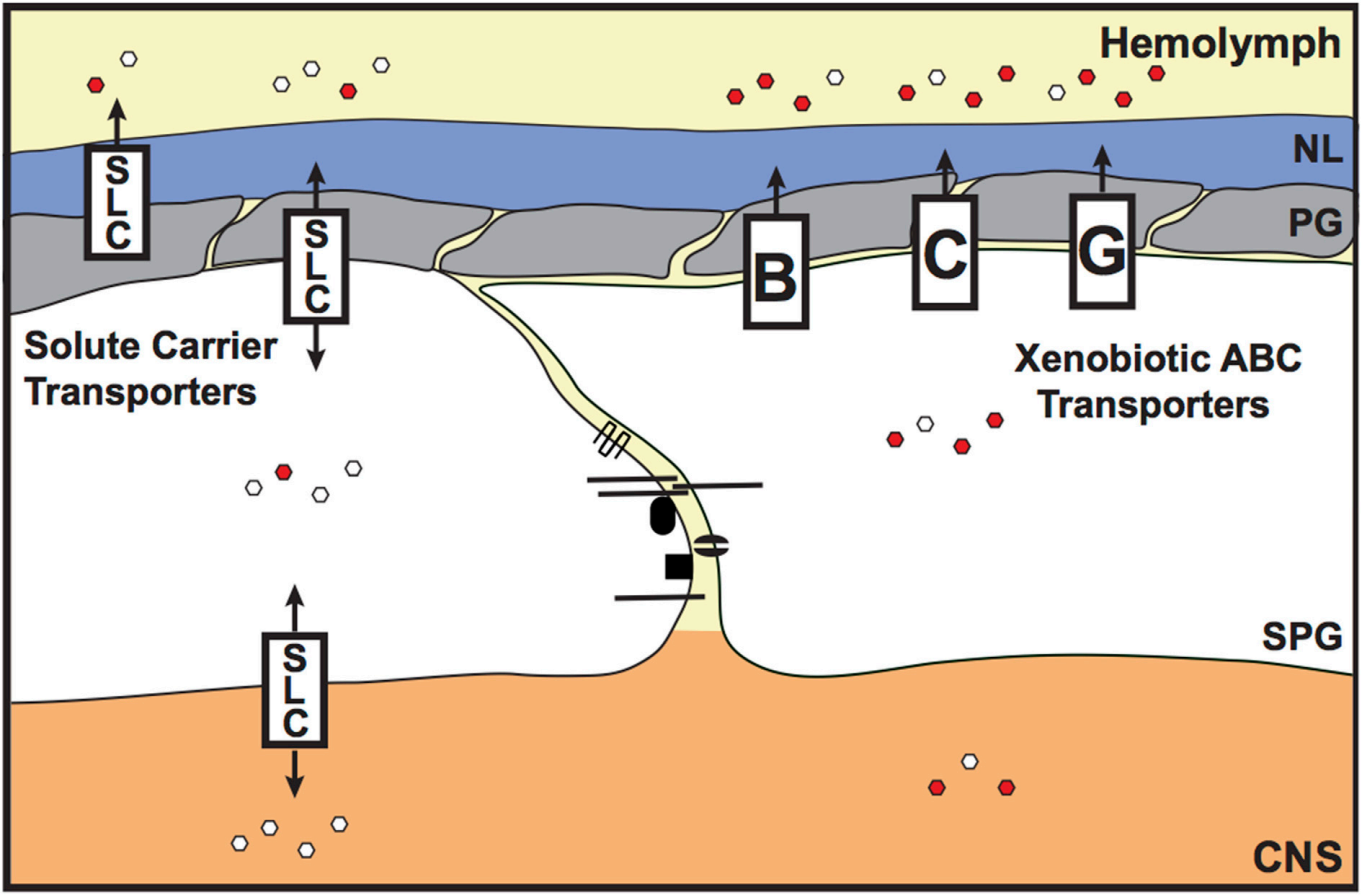
**Chemoprotective and endogenous molecule transporters of the invertebrate BBB**. The transcellular chemoprotective barrier is primarily provided by the ATP-binding Cassette (ABC) transporters. These xenobiotic efflux transporters are localized to the apical surface of the subperineurial glia (SPG) and remove lipophilic molecules from the SPG membrane, preventing access to the brain. Members of the Solute Carrier (SLC) class of transporters also have some chemoprotective roles, but are mainly known as transporters of endogenous molecules. The SLCs control the entry of hydrophilic/charged molecules (e.g., glucose, amino acids and ions) into the brain, to support optimal brain function. SLCs are present both on the apical and basal surfaces of the SPG cells. Some SLC members, e.g., Oatp58Dc, are also expressed in the perineurial glia (PG). Red and white hexagons represent xenobiotics and endogenous molecules, respectively. NL, neural lamella; CNS, central nervous system.

In the vertebrate vascular endothelium, the tight junctions provide chemoprotection to the brain by preventing the entry of molecules via the paracellular route. However, another mechanism is required to prevent transcellular access to the brain. This is provided by the xenobiotic efflux transporters (Figure 4). These transporters belong to the ATP-binding cassette (ABC) B, C, and G classes, and are localized to the apical membrane of the vascular endothelium. The xenobiotic efflux transporters are well-characterized for their role in removing lipophilic xenobiotics from the plasma membrane (Leslie et al., 2005), preventing access into the brain. Hence, these transporters are a major hindrance to the successful delivery of therapeutics into the brain (Abbott and Romero, 1996; Abbott, 2013). The main xenobiotic efflux transporters at the BBB are P-glycoprotein (P-gp/ABCB1/Mdr1) and Breast Cancer Resistance Protein (BCRP/ABCG2). In invertebrates a transcellular transport-barrier has been identified in the SPG cells, which is provided by the homolog of P-glycoprotein (Mdr65; Mayer et al., 2009). *Drosophila* Mdr65 shares 42% sequence identity with P-glycoprotein. In the *Drosophila* brain, Mdr65 expression is restricted to the SPG layer of the BBB and, like P-glycoprotein, localizes to the apical membrane. Mayer et al. (2009) identified Mdr65 in a screen for increased BBB permeability to the P-glycoprotein substrate Rhodamine B. Mdr65 is also required at the BBB to protect the brain from various P-glycoprotein xenobiotic substrates, and sensitivity of the *Mdr65* mutants to these xenobiotics can be rescued by expression of human P-glycoprotein specifically in the SPG cells, confirming that Mdr65 provides an evolutionarily conserved chemoprotective transport barrier at the invertebrate BBB. Interestingly, recent work in the migratory locust (*Locusta migratoria*) and the desert locust (*Schistocerca gregaria*) has shown that P-glycoprotein-like xenobiotic transporters also provide a transcellular barrier in the locust brain (Nielsen et al., 2011; Andersson et al., 2013). These findings advance the possibilities of using insect models to study the roles of xenobiotic efflux transporters at the BBB (see below).

In addition to Mdr65, other potential homologs of mammalian chemoprotective ABC transporters are expressed at the invertebrate BBB (DeSalvo et al., 2014). These candidates were identified based on their expression or enrichment in the adult *Drosophila* BBB; these include putative homologs for *ABCC-/MRP-1, 4, 5* and *6* (*CG5789, SUR, CG11897*, and *MRP*) and *ABCG2/BCRP* (*W, CG31689* and *CG3164*). Currently, there are no published reports showing whether these gene products can perform chemoprotective functions at the invertebrate BBB.

The vertebrate organic anion transporters (Oats and Oatps) are primarily known for their roles in controlling the brain access of endogenous anions (Kusuhara and Sugiyama, 2005); however, they also have chemoprotective functions (Anderson and Thwaites, 2010). Potential homologs of vertebrate Oats and Oatps are also expressed at the *Drosophila* BBB (*CG6126, CG3168, CG6231, ORCT2, CG4630, ORCT, Oatp58Dc*, and *Oatp74D*; DeSalvo et al., 2014), providing candidates for determining whether Oat/Oatps can also contribute toward chemoprotection of the brain in invertebrates. The *Drosophila* genome encodes eight Oatp transporters, two of which have enriched transcript number in the *Drosophila* brain (*Oatp58Dc* and *Oatp74D*; http://flyatlas.org/). Seabrooke and O'Donnell (2013) have recently shown that Oatp58Dc is expressed in the PG, SPG and post-mitotic neurons, and is required specifically in PG cells for maintaining chemical protection of the brain from fluorescein. In the absence of Oatp58Dc, levels of hemolymph-injected fluorescein are higher in the brains of *Oatp58Dc Drosophila* brains. This shows that, at least for Oatp58Dc, invertebrate organic anion transporters can also provide chemoprotective functions to the BBB.

### Endogenous molecule regulation

Transcellular access to the brain is not only controlled by ATP-dependent xenobiotic efflux transporters; secondary/facilitated transporters and channel proteins are also expressed at the BBB in both vertebrates and invertebrates (Figure 4). The largest group of the facilitated transporters is the solute carrier (SLC) transporters. In vertebrates, the SLCs are grouped into 48 classes (SLC 1–48) based on sequence identity (Hediger et al., 2004). It was recently documented that *Drosophila* glia express SLCs from eight of these vertebrate SLC classes (*SLC1, SLC5, SLC6, SLC7, SLC24*, and *SLC29*), but it was not determined whether these SLCs were expressed or enriched in the BBB (Featherstone, 2011). Microarray analysis of the adult *Drosophila* BBB glia has recently identified which of these SLCs are expressed or enriched at the BBB, and also identified additional BBB SLC class candidates (DeSalvo et al., 2014). Of note, there are 18 SLC homologs that are both highly expressed and enriched at the *Drosophila* BBB (members from the SLCO, 2, 7, 16, 19, 22, and 39 classes) and would therefore be good candidates for further study.

A role for the SLC12 transporter Ncc69 (a cation chloride cotransporter) and its serine/threonine kinase regulator Fray has been shown in controlling extracellular volume in peripheral nerves (Leiserson et al., 2011). Mutants in either *Ncc69* or *Fray* lead to the accumulation of fluid between axons and glia, resulting in peripheral neuropathy. Both Ncc69 and Fray were also shown to function in the SPG cells of the blood-nerve barrier.

Eight potential SLC21 (Oatp) transporters have been identified in *Drosophila* (Seabrooke and O'Donnell, 2013). Of these eight, four are expressed in the *Drosophila* head or brain (*Oatp30B, Oatp33Ea, Oatp58Dc*, and *Oatp74D*; http://flyatlas.org/; Torrie et al., 2004; Seabrooke and O'Donnell, 2013). According to the data in DeSalvo et al. (2014), *Oatp30B* is primarily expressed in neurons, however, Meyer et al. (2014) suggested a glial role for Oatp30B in neural lamella integrity and CNS shape. *Oatp33Ea* shows very low expression in all cell types of the adult brain; as its expression was previously shown in the fly head, Oatp33Ea may therefore localize to the fat bodies of the head rather than the brain. As mentioned above, the organic anion transporter Oatp58Dc is expressed in the PG, SPG and post-mitotic neurons of the *Drosophila* CNS; however, its function was shown to be specifically required in the PG layer of the BBB (Seabrooke and O'Donnell, 2013). This suggests a novel role for the PG in regulating organic anion access to the brain. *Oatp74D* was also shown by DeSalvo et al. (2014) to be highly enriched in the BBB (21-fold and 64-fold enrichment compared to whole brain and neuron levels, respectively). It would be interesting to determine the function of this transporter, whether it is required in the SPG or PG, and whether there is any overlap or coordination in the BBB functions of Oatp58Dc and Oatp74D.

Although the BBB provides a paracellular barrier to the movement of inorganic ions between the hemolymph and the brain, transcellular transit does occur (Treherne, 1966). This involves both energy-dependent mechanisms (Na^+^/K^+^ ATPases) and also diffusion down the electrochemical gradient, which may be partially set up by V-type H^+^-ATPases (Kocmarek and O'Donnell, 2011).

Another mode of transit across the BBB is provided by receptor-mediated transcytosis. In general, vesicular transcytosis is reduced in the vascular endothelial cells of the vertebrate BBB; however, small and large molecular weight molecules can transit the BBB by a specific, receptor-mediated route (see Xiao and Gan, 2013 for review). In *Drosophila*, the mechanism of receptor-mediated transcytosis of lipoprotein via the low-density lipoprotein receptor (LRP) has recently been investigated (Brankatschk and Eaton, 2010; Brankatschk et al., 2014). Lipophorin is the main lipoprotein present in *Drosophila*. The protein component of lipophorin (apolipophorin) can be cleaved into ApoLI and ApoLII, and this cleavage was shown to affect the localization of the lipoprotein once it had transited across the BBB. The full length ApoL localized to neurons, whereas ApoLII was found in the optic anlage. The authors further investigated the function of lipophorin in the brain and found that knocking down the production of lipophorin in its synthesis organ (the fat body) blocked neuroblast proliferation in the first/second instar larvae. They suggest that this may be due to the reduced BBB transit of GPI-linked proteins, which they show requires the presence of lipophorin. Brankatschk and Eaton (2010) also showed that lipophorin-associated, sterol-linked fluorescent proteins only have limited access to the brain and primarily accumulate at the BBB. From this, one might conclude that sterol-linked proteins don't play a role in neuroblast proliferation as they scarcely enter the brain; however, recent work in the Brand lab discovered a role for synchronized BBB calcium signaling in coordinated neuroblast proliferation (Speder and Brand, 2014; see below). Therefore, sterol-linked proteins may not need to enter the brain to induce neuroblast proliferation. Instead their accumulation in the BBB may trigger a signaling cascade that induces the underlying neuroblasts to proliferate. Brankatschk et al. (2014) have also shown that BBB calcium release is required for the BBB transit and normal brain localization of Lipid Transfer Particle. This was shown to be important for the insulin-dependent regulation of systemic growth (see below).

### Metabolic and signaling roles of the invertebrate BBB

**Figure 5:**
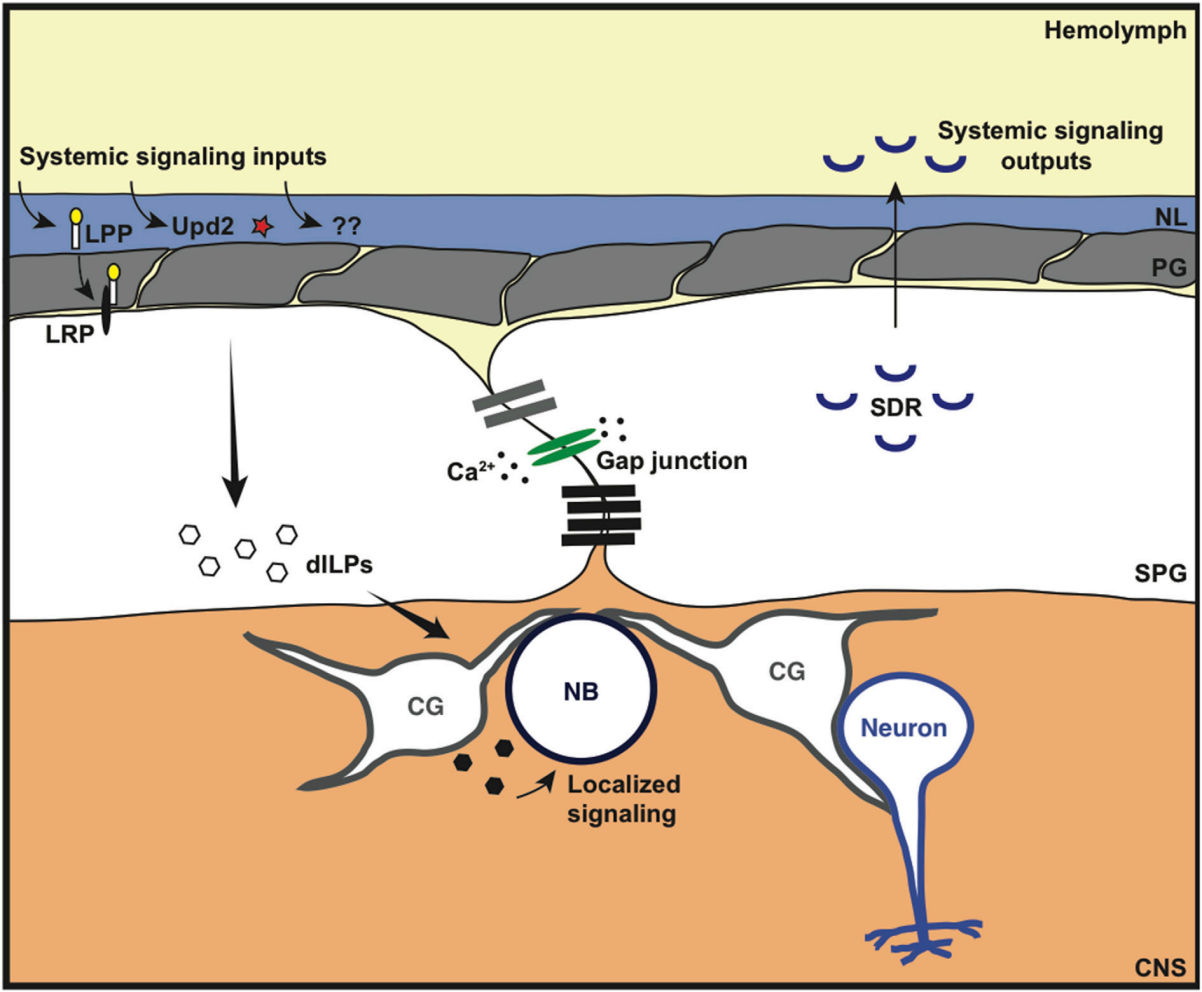
**Metabolic and signaling roles of the invertebrate BBB**. The *Drosophila* BBB can sense and respond to various systemic signaling inputs. This includes the lipoprotein-like receptor (LRP) ligand lipophorin (LPP), the cytokine-like molecule Unpaired-2 (Upd2) and other unidentified signaling molecules (??) that are released in response to metabolic/nutritional triggers. This leads to localized secretion of *Drosophila* insulin-like peptide (dILP) by the subperineurial glia (SPG) and/or cortex glia (CG) to coordinate neuroblast (NB) proliferation. The coordinated response of the SPG is thought to occur through synchronous calcium (Ca^2+^) oscillations via gap junction proteins. In addition to localized growth regulation, the *Drosophila* BBB can also regulate systemic growth (whole organism growth). The SPG cells both express and constitutively secrete a negative regulator of insulin signaling: the secreted decoy of insulin receptor (SDR); SDR mutants lead to excessive growth of the whole animal.

The previous sections have primarily dealt with the anatomy and function of the invertebrate BBB in chemoprotection of the brain, regulation of endogenous molecule access to the brain, and the maintenance of these functions. However, the BBB acts as more than just a selective filter. Exciting new findings have revealed that the *Drosophila* BBB can also sense and respond to systemic metabolic signals (Chell and Brand, 2010; Speder and Brand, 2014). Thus, the BBB appears to be a dynamic and communicative layer between the brain and the body (Figure 5). The insulin signaling pathway has long been known to sense nutritional status and dictate organism growth, reproductive output and lifespan, and this is evolutionarily conserved (Partridge et al., 2011; Wigby et al., 2011; Mirth and Shingleton, 2012). This systemic signaling is coordinated by the secretion of *Drosophila* insulin-like peptides (dILPs), which are secreted by insulin producing cells (IPCs) in the brain. The secretion of dILPs is triggered by a factor produced by the fat body (the *Drosophila* liver and adipose tissue equivalent) in response to dietary amino acids [through the Slimfast and target of rapamycin (TOR) signaling pathways; Geminard et al., 2009], and fat and sugar (through the *Drosophila* cytokine Unpaired2; Rajan and Perrimon, 2012; reviewed in Nassel et al., 2013). In addition to the IPCs, the BBB glia have recently been shown to be a source of dILPs required for the reactivation of proliferation in the underlying neuroblasts (Chell and Brand, 2010; Speder and Brand, 2014; Figure 5). The BBB glia expression of dILP2 and dILP6 is nutrient-dependent, suggesting that a humoral signal from the fat body (possibly Unpaired2; Rajan and Perrimon, 2012) is sensed by the surface glia leading to dILP production and secretion to reactivate neuroblast proliferation in the larval brain (Chell and Brand, 2010). It would be interesting to determine whether this humoral signal is the same signal that depends upon lipophorin particles to induce neuroblast proliferation, as shown by Brankatschk and Eaton (2010).

Additional evidence for the role of BBB glia in coordinating neuroblast proliferation was provided by Speder and Brand (2014) who discovered that gap junctional proteins expressed in the SPG cells were required for synchronized calcium oscillations within the SPG, and for neuroblast proliferation. The synchronized calcium oscillations were nutrient-dependent, and were therefore suggested to be a bridge in the gap between the nutrition-dependent fat body-derived signal and the synchronous nutrient-dependent exit of neuroblasts from quiescence. Although this work suggests a direct role for the BBB glia in neuroblast proliferation, Sousa-Nunes et al. (2011) suggest that neuroblast proliferation requires dILP6 expression by the underlying cortex glia rather than the SPG cells. Therefore, the role of the BBB may be to coordinate a complex signaling network, bringing together systemic nutritional signals and relaying this information to the underlying cortex glia to regulate neuroblast proliferation (Figure 5).

In extension of this work, Brankatschk et al. (2014) have shown that the BBB can also regulate growth systemically (whole organism growth) in addition to localized growth control (neuroblast proliferation). They found that the BBB controls Lipid Transfer Particle brain access and localization to Dilp2-recruiting neurons in the brain; this resulted in systemic secretion of Dilp2 from insulin producing cells in the brain, impacting on whole organism growth. This BBB function is nutrition-dependent and requires calcium signaling in the BBB. In fact, genetic manipulations that forced an increase in BBB calcium release resulted in normal systemic insulin signaling despite an inappropriate nutritional state. Together, these fascinating findings show that the BBB can have a master role in linking nutritional input to both local and systemic growth outputs.

A further role for the *Drosophila* BBB in growth control was shown by Okamoto et al. (2013). The authors discovered a negative regulator of insulin signaling, the secreted decoy of insulin receptor (SDR), in an RNAi screen for secreted regulators of body growth; *SDR* mutants had a larger body weight. SDR is expressed in the BBB glia of the CNS, the midgut muscles and imaginal discs of the larva; however, its function in body growth was shown to be dependent on its BBB glia expression (Okamoto et al., 2013). Although SDR expression and secretion was shown to be constitutive, and not dependent on nutritional status, it proved to be crucial for controlling body weight during adverse nutritional conditions; *SDR* mutants were lethal when grown in adverse nutritional conditions whereas wild type larvae survived. These results suggest a further role for the BBB in controlling body growth during development. The body size of the adult fly is predetermined during the larval and pupal stages (Mirth and Shingleton, 2012); however, DeSalvo et al. (2014) showed that *SDR* is highly expressed and enriched in the BBB glia of the adult brain. Therefore, this begs a number of questions. What is the role of SDR in the adult? Is its secretion regulated or constitutive in the adult? Does its expression level change with age? Does SDR have a role in regulating aging? These are exciting questions that can easily be addressed in the *Drosophila* model organism.

This additional role of the BBB in coordinating both localized and systemic insulin signaling to regulate localized and organism-wide growth is likely to be one in a number of ways that the BBB can communicate with various organs of the body.

## The invertebrate BBB as a tool for advancing therapeutic interventions

One of the major hindrances in neuropharmacology is the resistance of the BBB for drug delivery to the brain. This is primarily due to the efficient functioning of P-glycoprotein. Furthermore, the pre-clinical to clinical translation of CNS drug penetration following the use of P-glycoprotein inhibitors has proved unsuccessful due to incomplete P-glycoprotein inhibition (see Kalvass et al., 2013 for a detailed review). Therefore, being able to develop drugs that are not P-glycoprotein substrates would be a beneficial strategy. Drug screening largely depends upon *in vitro* tests using brain microvascular endothelial cell monolayers. However, studies have shown that brain microvascular endothelial cells grown in culture do not maintain their full BBB properties; of particular note, they show a down regulation of P-glycoprotein (Calabria and Shusta, 2008), suggesting that this methodology is not ideal. Early phase drug screening using rodents would be too costly; therefore, there is a need for a cost-effective *in vivo* approach to further the development of CNS drugs. With this in mind, DeSalvo et al. (2011) developed an approach to perform live screening of chemical fluor penetration into the *Drosophila* brain. This approach involved visualizing a change in the fluorescence intensity in the fly eye, due to a change in permeability of the blood-eye barrier. DeSalvo et al. (2011) showed this approach could be used to screen for genetic modifiers of BBB integrity and identified various interesting candidate genes that were expressed in different layers of the compound BBB, supporting the notion that all layers of the compound barrier are important for full BBB integrity. In addition to genetic modifiers, this live imaging approach can be used for large-scale drug screening to identify drugs that will penetrate the BBB and are therefore not likely P-glycoprotein (Mdr65) substrates.

Interestingly, other invertebrate BBB models have been developed to allow *in vivo* drug screening. Using the grasshopper system, Nielsen et al. (2011) showed that co-administration of a P-glycoprotein inhibitor with a test P-glycoprotein substrate rendered the BBB more permeable to the P-glycoprotein substrate. Furthermore, Andersson et al. (2013) were able to use this methodology to distinguish P-glycoprotein substrates from non-substrates using liquid chromatography-mass spectrometry to measure the drug content in the brain, either in the absence or presence of a P-glycoprotein inhibitor. This confirmed that P-glycoprotein substrates could be identified using this model system. These invertebrate BBB models provide a valuable bridge between endothelial cell culture models and vertebrate P-glycoprotein knock-out models for drug screening.

In addition to using invertebrate BBB models to assess BBB permeability of test drugs, these model systems can also be used to test new technologies for increasing drug delivery to the brain. The cost of performing these trials in invertebrates would be comparatively small and would therefore allow for a much broader scaled testing strategy than can be applied to vertebrate systems. The use of *Drosophila* to address the problem of brain drug delivery was shown by Sarantseva et al. (2011), where they tested the ability of dendrimers to cross the BBB. Dendrimers are nanopolymers that can encapsulate various cargo and therefore have been utilized in many biological (and non-biological) fields (see Kesharwani et al., 2014 for a review). The advantage of using these nanopolymers as a CNS drug delivery strategy is the ability to customize the dendrimer to very specific requirements. For example, the functional group on the polymer surface can be modified to allow tissue-specific targeting, and they are also able to “carry” various types of cargo, including nucleic acids, proteins and drugs across cell membranes (Kesharwani et al., 2014). The use of these biological vectors in drug delivery have been shown for epithelial barriers, such as the skin and intestine (e.g., Kaminskas et al., 2012). Moreover, the ability of the lysine-based dendrimer D5 to carry peptides across the *Drosophila* BBB has been shown (Sarantseva et al., 2011), suggesting that this nanotechnology could be a useful strategy for improving CNS drug delivery in vertebrates.

## Future questions and emerging areas of BBB research

We have highlighted that the invertebrate BBB is a compound structure, like the vertebrate neurovascular unit, and each of its cellular and non-cellular layers appears to have a coordinated role in maintaining BBB integrity and for signaling purposes. However, there is still much to learn about the nature of the communication between these layers, how this is regulated, and how the individual layers respond to physical, chemical or pathological perturbations. In particular, understanding the compensations that occur within these BBB layers during a disease state will likely be required to advance therapeutic design to combat these disorders. Expression changes of chemoprotective transporters, such as P-glycoprotein and Breast Cancer Resistance Protein, have already been shown to occur in disease states including Alzheimer's disease and epilepsy (Vogelgesang et al., 2002; Loscher and Potschka, 2005; Miller, 2010). These expression changes are thought to explain the multidrug resistance phenotypes seen in some neurological disorders. Therefore, investigations that show ways to counter these compensations would be valuable for treating these diseases. Furthermore, being able to parse the contribution of the PG and SPG toward CNS protection would be a valuable endeavor. This could involve a genetic approach, like used by DeSalvo et al. (2014), to produce separate PG and SPG transcriptomes in both the wild type and diseased states.

In addition to parsing the contributions of the SPG and PG toward BBB function, an important future question for the field is to determine whether there are regional specializations across the invertebrate compound BBB. We have seen evidence of coordinated BBB functions via gap junction signaling within the SPG cells, which impacted upon the synchrony of neuroblast proliferation (Speder and Brand, 2014); equally possible is the presence of regional BBB responses to localized signals, in terms of developmental and pathological signals, as well as localized immune cell transmigration. In extension of this, it is important to understand how the different layers of the compound BBB network can respond to localized signals and perturbations. For example, it would be interesting to determine whether there are different subsets of underlying cortex glia that can sense and respond to chemical perturbations of the BBB and other subsets that can respond to the metabolic status of the CNS, to provide both broad and localized “feedback circuitry” to relay information about CNS homeostasis to the BBB. Due to the powerful genetic strengths of the *Drosophila* system and the accessible architecture of the invertebrate BBB, this is an area where invertebrate model organisms can help to pave the way forward.

An exciting, emerging area of BBB research that deserves further attention is whether there are sex-specific traits of the BBB. It is already known that there are sex-specific differences in the CNS, a concept that is evolutionarily conserved (Cachero et al., 2010). However, very little is known about sex-specific components of the BBB and its impact on physiology. This is another research area where *Drosophila* can advance the field, as it is possible to specifically change the sex of the BBB using targeted genetics. This allows the separation of sex-specific BBB effects from sex-specific effects of the whole organism. This approach is possible because sex determination is cell autonomous in flies; therefore, one can express a master regulator of sex determination (e.g., *TraF*) in a specific cell type and alter the sex identity of that cell type. This has been shown for the *Drosophila* liver and adipose tissue equivalent (the fat body), showing that male courtship behavior can be altered by changing the sex identity of the fat body cells (Lazareva et al., 2007). This technique was recently applied to the BBB, showing a behavioral impact of sex-specificity in the BBB (Hoxha et al., 2013). BBB-specific, ectopic expression of a gene that feminizes cells (*TraF*) led to reduced courtship behavior in males. Hoxha et al. (2013) showed that this was not solely a developmental effect, as BBB-specific expression of TraF only during the adult stages of the fly still reduced courtship in males. The authors also showed that this reduction in courtship behavior required the GPCR Moody, which Bainton et al. (2005) previously showed to have separable roles in maintaining BBB integrity (a structural role) and behavioral drug responses (a signaling role). Interestingly, there are sex-specific enrichments of *Moody* isoforms in the BBB (Hoxha et al., 2013). Of the four *Moody* isoforms, there is an enrichment of one of the *Moody*-α isoforms in male heads and an enrichment of both *Moody*-β isoforms in female heads. Therefore, Hoxha et al. (2013) have already identified that sex-specific transcripts do exist in the BBB and that sex identity of the BBB can impact upon behavior. It would be interesting to determine whether sex-specific differences in the BBB can also impact upon pharmacokinetics, as this could have major implications for treatments of CNS disorders.

### Conflict of interest statement

The authors declare that the research was conducted in the absence of any commercial or financial relationships that could be construed as a potential conflict of interest.

## Abbreviations

BBB: blood-brain barrier
SPG: subperineurial glia
PG: perineurial glia
CNS: central nervous system
ABC: ATP-binding cassette
SLC: solute carrier
GPCR: G-protein coupled receptor
ARSs: actin-rich structures
IPCs: insulin producing cells
dILPs: *Drosophila* insulin-like peptides
